# Application of a novel three-day repetitive transcranial magnetic stimulation protocol for the treatment of drug-resistant epilepsy in dogs: single-blinded randomised sham-controlled clinical trial

**DOI:** 10.3389/fvets.2025.1598311

**Published:** 2025-08-06

**Authors:** Marios Charalambous, Delia Hünting, Nina Meyerhoff, Friederike Twele, Sebastian Meller, Holger A. Volk

**Affiliations:** ^1^Small Animal Department, University of Veterinary Medicine Hannover, Hannover, Germany; ^2^Center for Systems Neuroscience Hannover, Hannover, Germany

**Keywords:** seizures, dogs, refractory, management, neurostimulation

## Abstract

While the efficacy of repetitive transcranial magnetic stimulation (rTMS) has been explored in humans and, to a lesser extent, in dogs with epilepsy, further clinical studies are required to assess the potential antiseizure effect of this non-invasive neurostimulation technique. The objective was to assess the antiseizure effect and safety of a novel three-day rTMS protocol in dogs with drug-resistant idiopathic epilepsy. A single-blinded, randomized, sham-controlled clinical trial was conducted by randomly allocating 20 dogs with drug-resistant idiopathic epilepsy or epilepsy of unknown origin into active (*n* = 10) or sham (*n* = 10) rTMS. The monthly seizure frequency (MSF), monthly seizure day frequency (MSDF), and number of cluster seizures (CS) were recorded and comparisons between the two groups were analysed. The safety of the rTMS protocol was also evaluated. Statistically significant differences were identified between the groups in median MSF (active, 8 [0–24]; sham, 17 [7–46]; *p* = 0.04), MSDF (active, 8 [0–24]; sham, 11 [6–23]; *p* = 0.04), and number of CS (active, 10 [5–23]; sham, 16 [10–25]; *p* = 0.005). No adverse events were reported. The current protocol indicates that active rTMS is safe, can reduce seizure frequency, and prevent CS in dogs with drug-resistant idiopathic epilepsy or epilepsy of unknown origin. An “one-size-fits-all” rTMS protocol for epilepsy in dogs is likely to provide suboptimal outcomes because the effect of rTMS is highly dependent on the duration and parameters of stimulation as well as individual variability. Therefore, future studies are needed to explore further specific stimulation parameters so they can be better tailored to the individual dog.

## 1 Introduction

Idiopathic epilepsy (IE) is a common neurological disorder in dogs, with an estimated prevalence ranging from 0.5 to 0.82% in the general population; in certain genetically predisposed breeds, this prevalence can be as high as 33% (1–8). Approximately 30% of dogs with IE exhibit drug resistance to multiple antiseizure medications (ASMs) (9), leading to poor prognosis and potentially contributing to the decision for euthanasia. Consequently, the evaluation and development of both pharmacological and non-pharmacological therapeutic options remains of highest priority in canine epileptology (10).

Non-invasive neurostimulation is an emerging therapeutic modality in veterinary neurology, involving techniques that can access and stimulate the nervous system without incising the overlying tissues. These methods, which include electrical, magnetic, or other forms of stimulation, aim to modulate the excitability of neural tissues and the broader neural networks involved. Transcranial magnetic stimulation (TMS) is a non-invasive neuromodulation technique based on the principle of electromagnetic induction, which generates an electric field within the brain (11). Initially, repetitive TMS (rTMS) was primarily used for diagnosing neuromotor disorders. However, one of its key therapeutic applications has emerged in the alteration of cortical excitability, providing an alternative treatment for brain and mental health disorders. In people, the FDA has approved multiple rTMS devices for the treatment of drug-resistant depression with or without anxiety disorder, obsessive-compulsive disorder, and substance use disorders, such as smoking cessation (12). Currently, rTMS devices are being used to manage various mental and neurological disorders in humans such as generalised anxiety disorder, migraine headache, Alzheimer’s disease, stroke, and chronic pain (13). Repetitive TMS devices are highly adaptable, allowing precise adjustment of various parameters, such as magnetic energy modulation in targeted cortical areas, to optimize treatment outcomes for various disorders in humans.

Over the past three decades, rTMS has garnered increasing attention from researchers as a potential therapeutic approach for epilepsy (10, 11, 13–15). TMS is a non-invasive, well-tolerated technique that modulates and stimulates the brain by generating small intracranial electrical currents through the application of a strong focused extracranial magnetic field. This method specifically targets the motor cortex (directly) and deeper networks (indirectly), and has shown promise in influencing neural activity relevant to epilepsy treatment (16). Given that epilepsy is characterized by altered neuronal networks leading to cortical hyperexcitability, there is strong justification for exploring the use of rTMS to reduce cortical excitability as a potential alternative to conventional treatments, particularly for drug-resistant epilepsy in humans and dogs, and epilepsy phenotypes, particularly when resective surgery of the epileptogenic zone(s) is not feasible (17, 18).

Repetitive TMS can deliver a series of TMS pulses at a constant intensity to a targeted brain region, with frequencies ranging from one stimulus per second to over 20 (10, 11, 19). It is well-established that the neurobiological effects of rTMS vary significantly across individuals (16). These effects are influenced by multiple factors, i.e., rTMS device-related, including stimulation frequency, stimulation intensity, number of trains and pulses within each train, type and position of the coil, and duration of stimulation as well as subject-related, including individual physiological and anatomical variability, and specific disorder targeted (11, 14, 15, 20, 21). Even though all of these parameter are crucial in determining the therapeutic outcome, coil intensity, stimulation frequency and duration of stimulation might be of particular consideration (10, 11, 14, 15). For instance, low frequency rTMS (LF-rTMS) provides an inhibitory effect which is both robust and long-lasting and can be applied to the motor cortex as well as other cortical regions (19, 22, 23). Optimizing stimulation parameters is crucial for the effective administration of rTMS due to its profound influence on neuronal network modulation.

A previous study by the primary author demonstrated that LF-rTMS administered over five consecutive days can serve as a safe and effective adjunctive treatment for dogs with drug-resistant IE (14). The objective of the current study was to evaluate the efficacy and safety of a modified LF-rTMS protocol applied over only three consecutive days in dogs with drug-resistant epilepsy.

## 2 Materials and methods

The study was reviewed and received ethical approval by the relevant German ethical committees in Lower Saxony (LAVES; reference number, 33.8–42,502–04-22-00114).

### 2.1 Population

Dogs diagnosed with drug-resistant epilepsy, irrespective of age, breed, or sex, were enrolled into the study. Dogs with seizure onset between 6 months and 6 years of age were classified as having IE, whereas those with onset outside this age range were classified as having epilepsy of unknown origin (EUO). For inclusion of EUO cases, a history of two or more unprovoked epileptic seizures occurring at least 24 h apart, normal neurological examination and unremarkable findings on diagnostic investigations—including laboratory tests, magnetic resonance imaging (MRI), and cerebrospinal fluid (CSF) analysis— were a requirement. Overall, the classification, definition, and diagnostic criteria for IE and EUO adhered to the guidelines outlined by the International Veterinary Epilepsy Task Force (IVETF) (24–26). When a particular ASM could no longer be titrated—either due to the attainment of maximum therapeutic serum levels or the emergence of unacceptable adverse effects—resistance to this ASM was assumed, and an additional agent was introduced. Drug-resistant epilepsy was defined as epilepsy demonstrating less than a 50% reduction in monthly seizure frequency (MSF), or a progressively increasing seizure frequency, despite treatment with at least two ASMs at optimal doses, serum drug concentrations, or both for an individual dog.

### 2.2 Sample size calculations

Sample size calculations were conducted based on data from a previously published study by the primary author (14). Estimation of the sample size was performed using the “G*Power” software. Specifically, we employed a t-test for the difference between two independent means (two groups). Based on the previous publication, the assumed means were 0.43 for group 1 and 0.99 for group 2, resulting in a calculated effect size (Cohen’s d) of 1.4. Based on this effect size, an alpha level of 0.05, and a statistical power of 80%, the required sample size was estimated to be 10 subjects per group. The calculations were also reviewed and approved by LAVES.

### 2.3 Procedure

The study was conducted as a single-blinded, randomized, sham-controlled clinical trial. The study included a pre-rTMS, rTMS and post-rTMS period. Specifically, a baseline epileptic seizure frequency, consisting of a three-month pre-treatment period, to assess baseline seizure frequency and the number of cluster seizures events (CS; defined as ≥2 epileptic seizures within a 24-h period) was recorded retrospectively. This was followed by daily administration of either active or sham (i.e., inactive; placebo) rTMS for three consecutive days. Finally, a three-month post-treatment period was evaluated to monitor seizure frequency and any adverse effects related to the treatment. During this time, owners maintained a seizure diary to document epileptic seizure frequency, type and severity and any potential treatment-related adverse effects. Dogs were randomly assigned to either the active or sham rTMS group using randomised envelopes. An equal number of entries indicating either active or sham rTMS was prepared and placed in the envelopes, which were sealed, mixed, and stored in a locked office for safety; then, they were randomly chosen for each enrolled dog. The investigator(s) were unaware of the randomization order. The owners, who were responsible for recording seizure frequency and adverse effects, were blinded to the treatment allocation.

**Figure 1:**
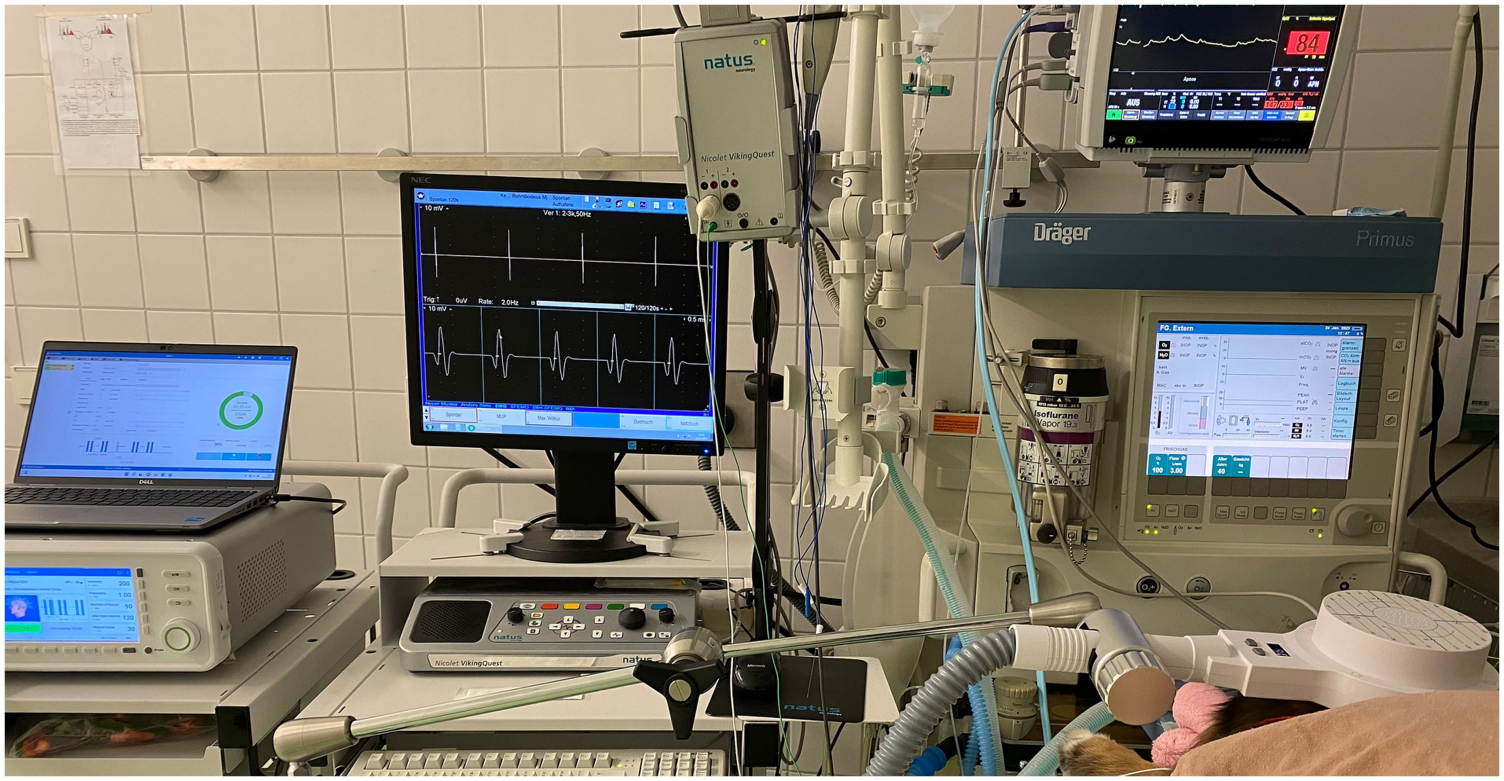
The arrangement of the medical equipment during active stimulation is displayed. From left to right: Magnetic stimulator, electromyography and anaesthetic monitoring device connected to the dog.

**Figure 2:**
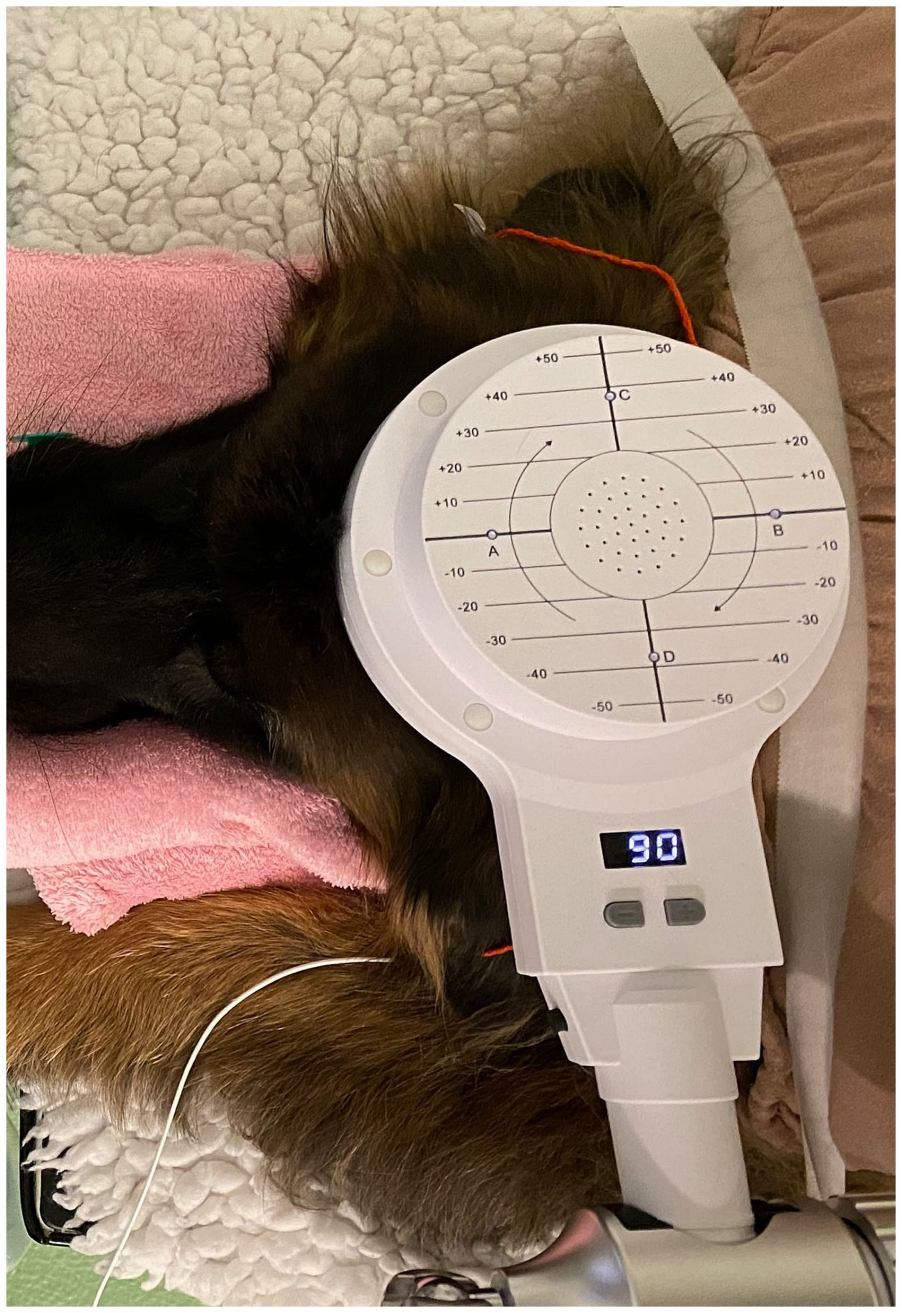
A dog with the coil parallel to and in contact to the vertex during active stimulation.

To facilitate sedation during the period of the rTMS treatment or sham procedure, intravenous catheters were inserted at the outset of the procedure without the presence of the owners. Blood samples for complete blood count, serum biochemistry, and ASM serum concentration assessment were collected from all dogs at that time. Dogs received daily active or sham rTMS, lasting 1 h and 40 min, over three consecutive days. Stimulation parameters and the study environment were consistent across both groups, with the sole distinction being that the sham group received inactive stimulation by positioning the round coil perpendicularly to and with a distance of 20 cm above the skull to prevent brain stimulation. All dogs in both groups were sedated with an intravenous bolus of dexmedetomidine (1 μg/kg), butorphanol (0.1 mg/kg) and midazolam (0.2 mg/kg) following catheter placement. They remained sedated throughout treatment via a constant rate infusion (CRI) of dexmedetomidine at 1 to 3 μg/kg/h. The duration of neurostimulation and, thus, sedation was equivalent for both groups. Crystalloid isotonic fluids (5 mL/kg/h) were administered in conjunction with the dexmedetomidine CRI during treatment, and butorphanol (0.1 mg/kg) was re-administered 1 h after the initial boluses. All dogs received oxygen at a flow rate of 2 L/min through an anaesthetic mask. Figure 1 represents the overall arrangement of the medical equipment utilised for the neurostimulation. Rubber earplugs were inserted into the dogs’ ears to mitigate potential noise disturbances from the rTMS machine during operation. The dogs were stabilized in ventral recumbency on the examination table using tape to minimize any minor movements. For dogs receiving active rTMS treatment, the round coil (with an outer diameter of 120 mm) was applied directly to the dog’s skull, centred at the vertex of the cranium (Figure 2).

The stimulation protocol involved 30 trains of 90 pulses each (total of 2,700 pulses) at LF (1 Hz, one pulse per second), with a 120-s inter-train interval. The coil output was set at intensity equal to 200% (i.e., double) of the output needed to reach the motor cortex threshold. The motor cortex threshold was established for each patient at the start of the treatment. This threshold was defined as the minimal TMS intensity (coil output) required to elicit at least five out of 10 electromyographic (EMG) responses (i.e., compound muscle action potentials [CMAPs] with an amplitude of at least 50 μV) from a fully relaxed thoracic limb muscle (external carpi radialis) (27). The CMAPs were recorded to ensure that the maximum coil output for each dog exceeds the motor cortex threshold (cortex stimulation occurs only if the coil output surpasses this threshold), as well as to monitor motor cortex stimulation during rTMS treatment and assess any fluctuations in the threshold (increase, decrease, or stable) during the active rTMS sessions.

During the post-rTMS period, the owners documented the epileptic seizure events and any potential adverse effects in a standardised seizure diary provided to them. No adjustments to ASM dosages were allowed in either group during the evaluation period; exception was the rescue ASMs, such as the short-term administration of benzodiazepines (via oral, intranasal, rectal, or intravenous routes) or levetiracetam pulse therapy (40–60 mg/kg administered orally or intravenously at once, followed by 20–40 mg/kg every 8 h until the dog remains seizure-free for 2 days, then discontinued) in cases of status epilepticus (i.e., continuous seizure activity lasting >5 min) or CS.

### 2.4 Data interpretation and statistical analysis

The following variables were recorded and assessed for each dog: MSF, monthly seizure day frequency (MSDF), and the monthly number of CS events. The MSDF was used with the aim to reduce the potential bias introduced by CS. Assessing the frequency of CS separately was essential, as these episodes represent a more critical form of seizure activity and have been linked to the development of drug-resistant epilepsy (10, 28); therefore, a reduction in their occurrence is considered a meaningful therapeutic success. Overall, prevention of emergent seizure patterns, such as CS and status epilepticus, is widely regarded as a clinically important and desirable outcome in the evaluation of antiseizure interventions. A multiple testing correction was not applied because the three outcome measures, i.e., MSF, MSDF, number of CS, employed in our study were all derived from the same underlying parameter—seizure frequency—and thus do not represent entirely distinct outcomes.

Pre-and post-rTMS values for MSF, MSDF, and monthly number of CS were compared between the sham and active rTMS group using the Mann–Whitney U test, with statistical significance defined as *p* ≤ 0.05. The MSF was also used to evaluate the duration of rTMS effectiveness in each dog treated with active rTMS. Specifically, the rTMS effect was considered to persist as long as the post-MSF remained above the pre-MSF level.

Data were analysed using an intention-to-treat (ITT) approach was implemented to account for potential early withdrawals from the study. The ITT approach assessed all the randomized subjects in the groups to which they were assigned, regardless of any subsequent withdrawal (29). Specifically, the ITT approach encompassed every subject randomized according to their treatment assignment while disregarding noncompliance, protocol deviations, withdrawals, and any events occurring post-randomization (30). This approach was used to mitigate overoptimistic estimates of intervention efficacy that may arise from excluding noncompliant participants, acknowledging that noncompliance and protocol deviations are common in clinical practice; overall, ITT approach can (i) maintain the integrity of randomization, (ii) minimise selection bias and bias associated with attrition or protocol deviations, (iii) provide a pragmatic estimate of treatment effectiveness that better reflects real-world clinical scenarios, (iv) reduce the risk of false-positive findings, and v) enhance the reliability and interpretability of the study conclusions (29, 30). For the ITT analysis, missing values were imputed as follows: if a dog withdrew from the study, their last recorded values were carried forward throughout the data analysis. Consequently, for dogs in the sham group, the missing value was set equal to the neighbouring value corresponding to better performance; for dogs in the active group, the missing value would correspond to the neighbouring value indicating worse performance. An alpha level of 0.05 was established for model significance. The software used was the GraphPad Prism version 10.4.1 (GraphPad Software, Inc., La Jolla, CA, USA).

## 3 Results

**Figure 3:**
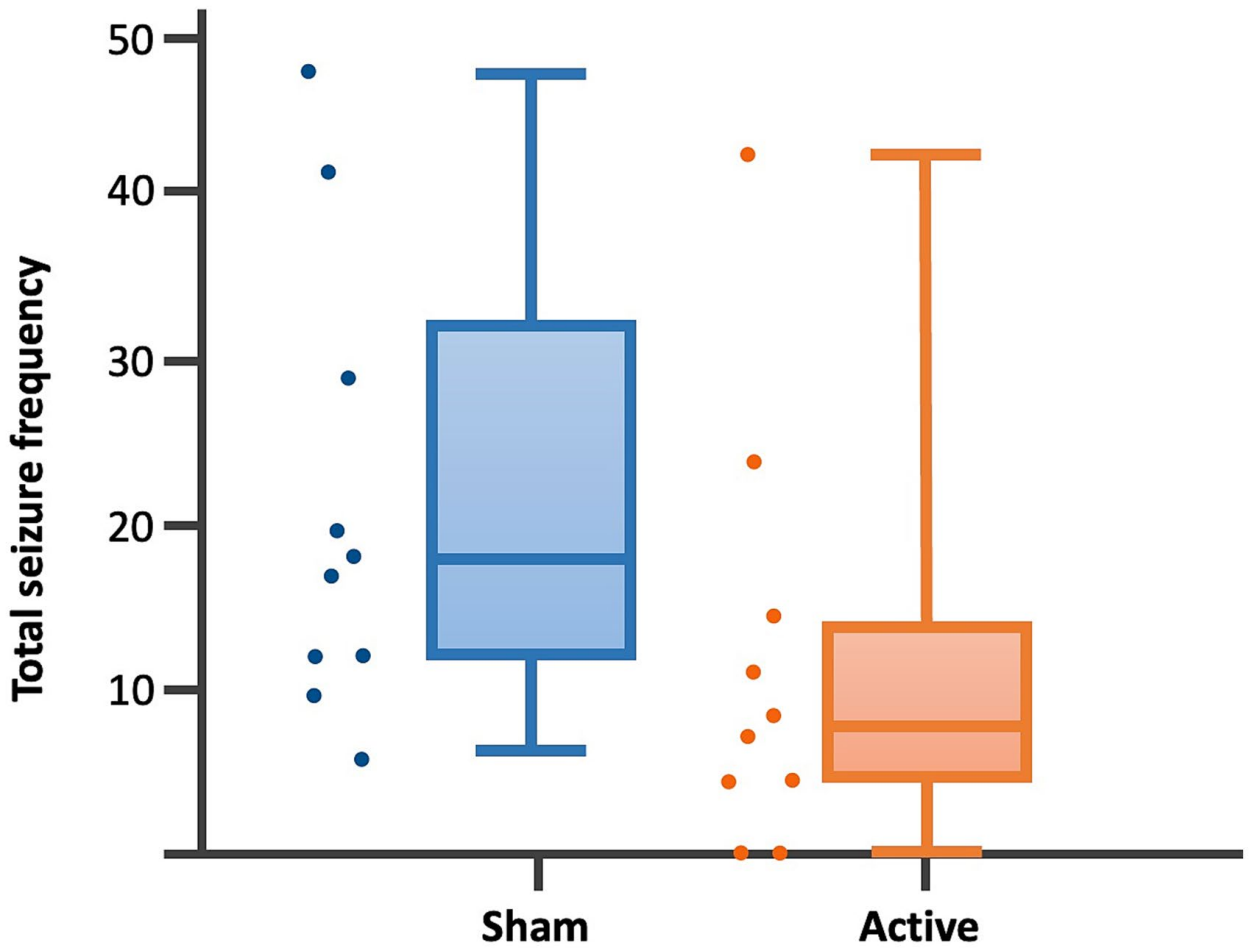
Dot-boxplots displaying the statistical comparison of post-rTMS total SF between active and sham groups. Mann Whitney-U-Test (*p* = 0.04).

**Figure 4:**
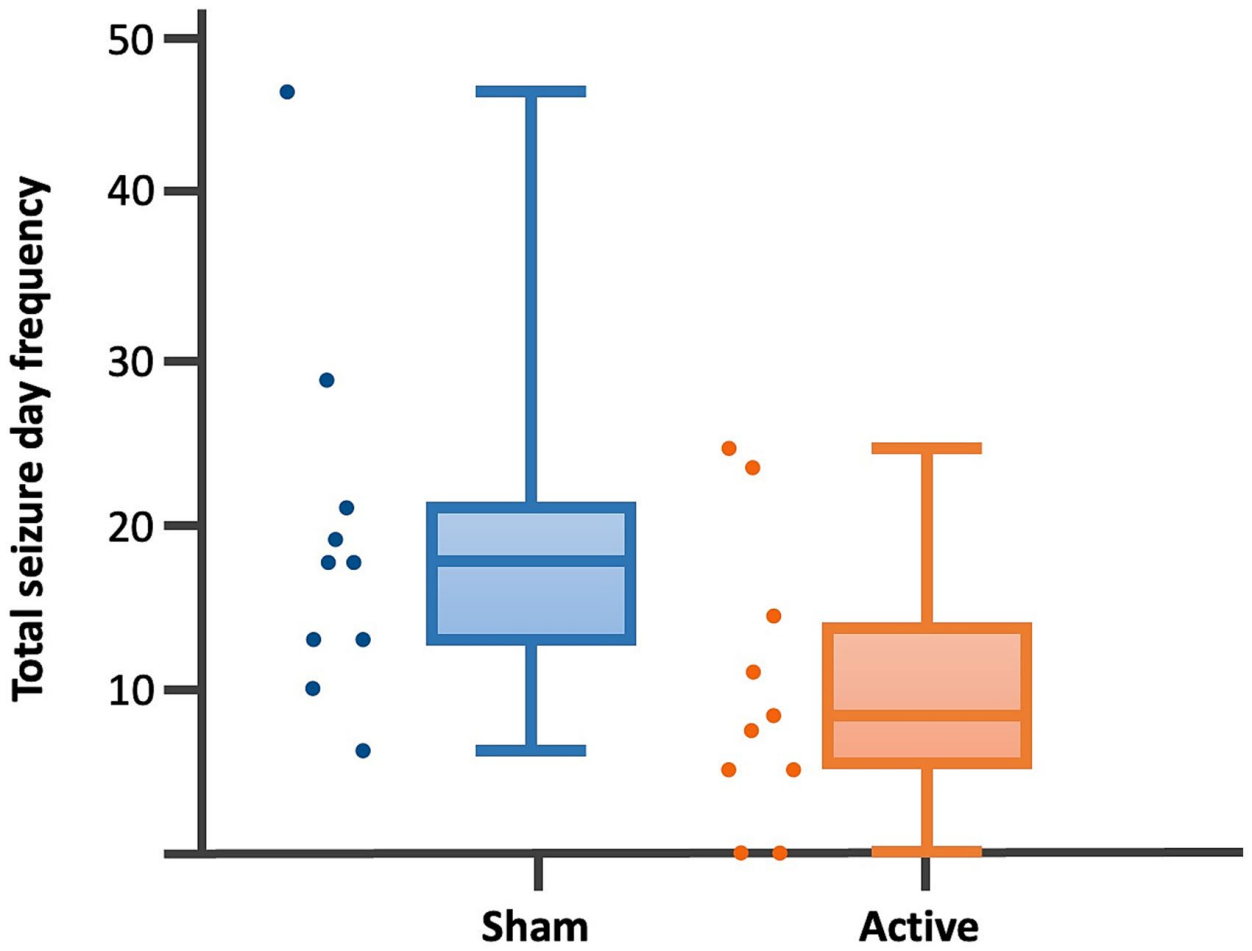
Dot-boxplots displaying the statistical comparison of post-rTMS total SDF between active and sham groups. Mann Whitney-U-Test (*p* = 0.04).

**Figure 5:**
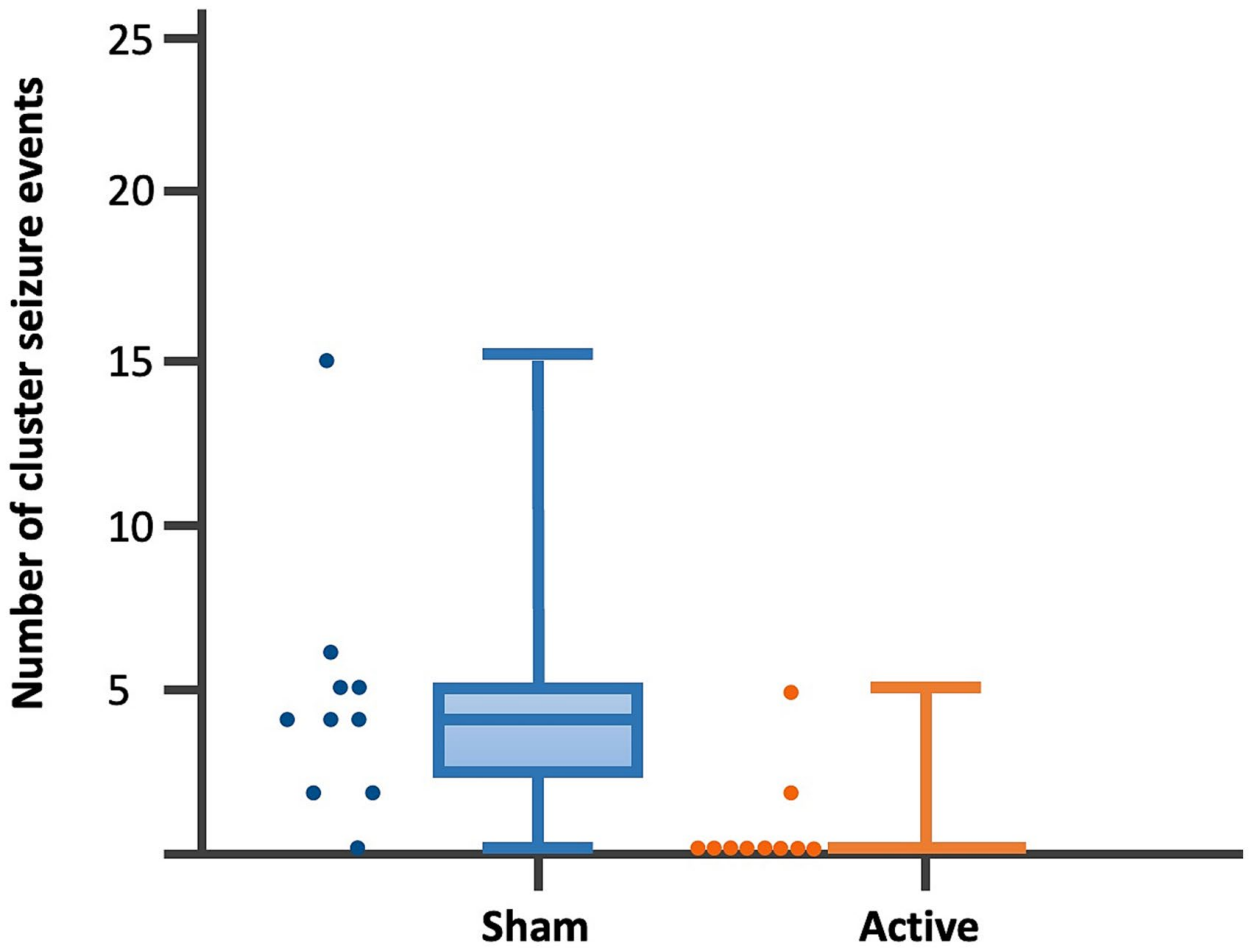
Dot-boxplots displaying the statistical comparison of post-rTMS number of CS events between active and sham groups. Mann Whitney-U-Test (*p* = 0.005).

**Figure 6:**
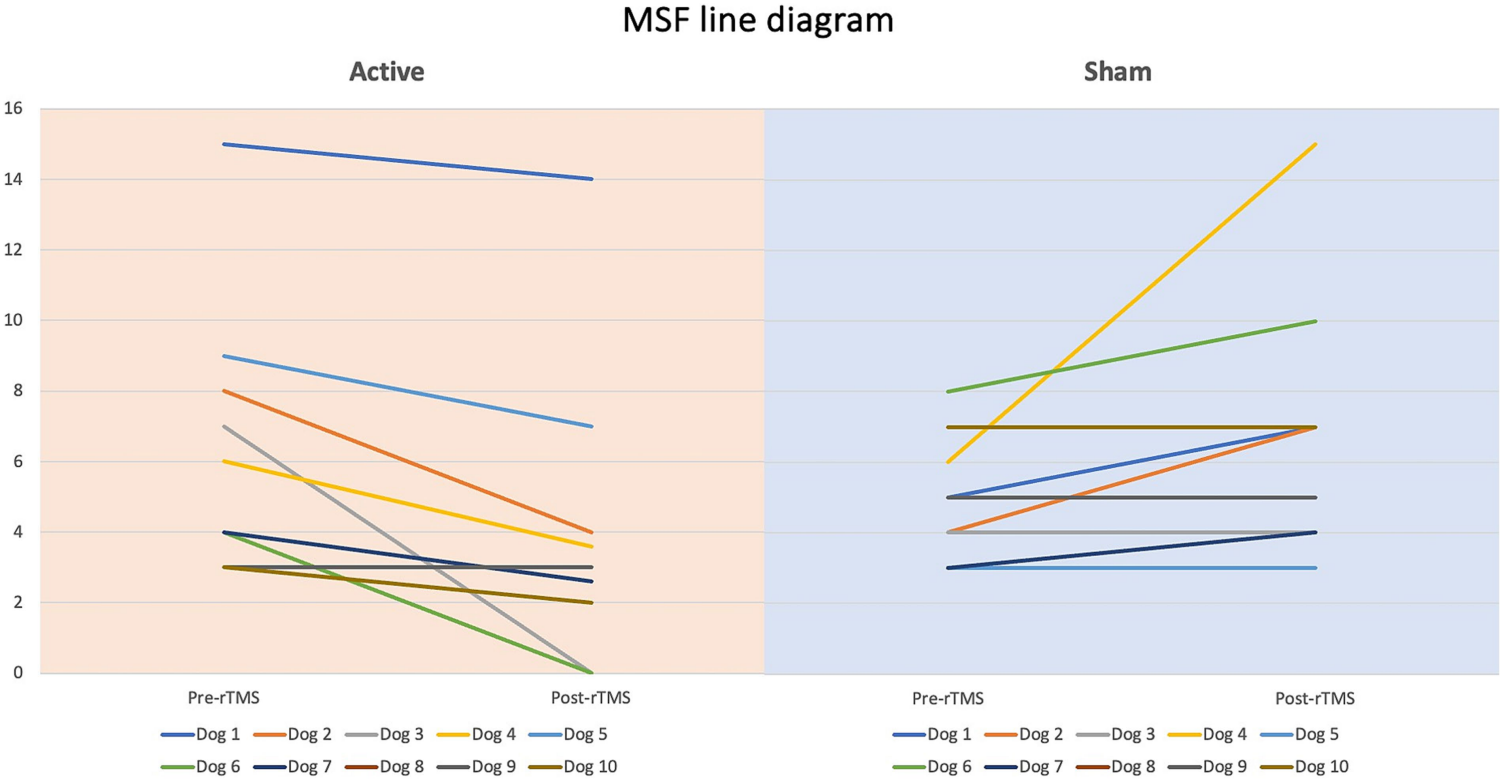
Line diagram showing the changes in MSF pre- vs. post-rTMS in both groups for each dog.

**Figure 7:**
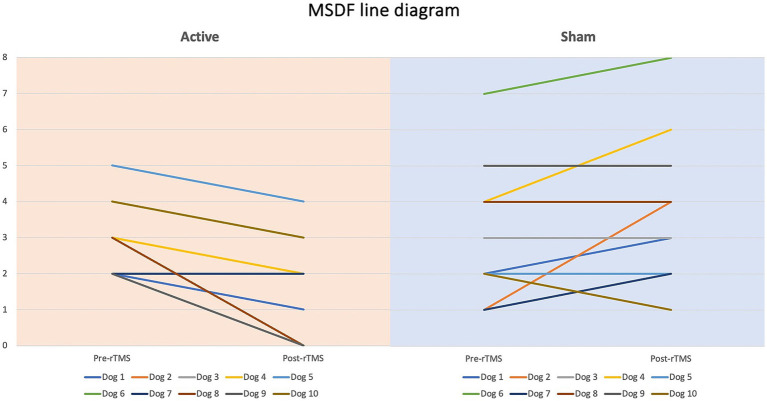
Line diagram showing the changes in MSDF pre- vs. post-rTMS in both groups for each dog.

**Figure 8:**
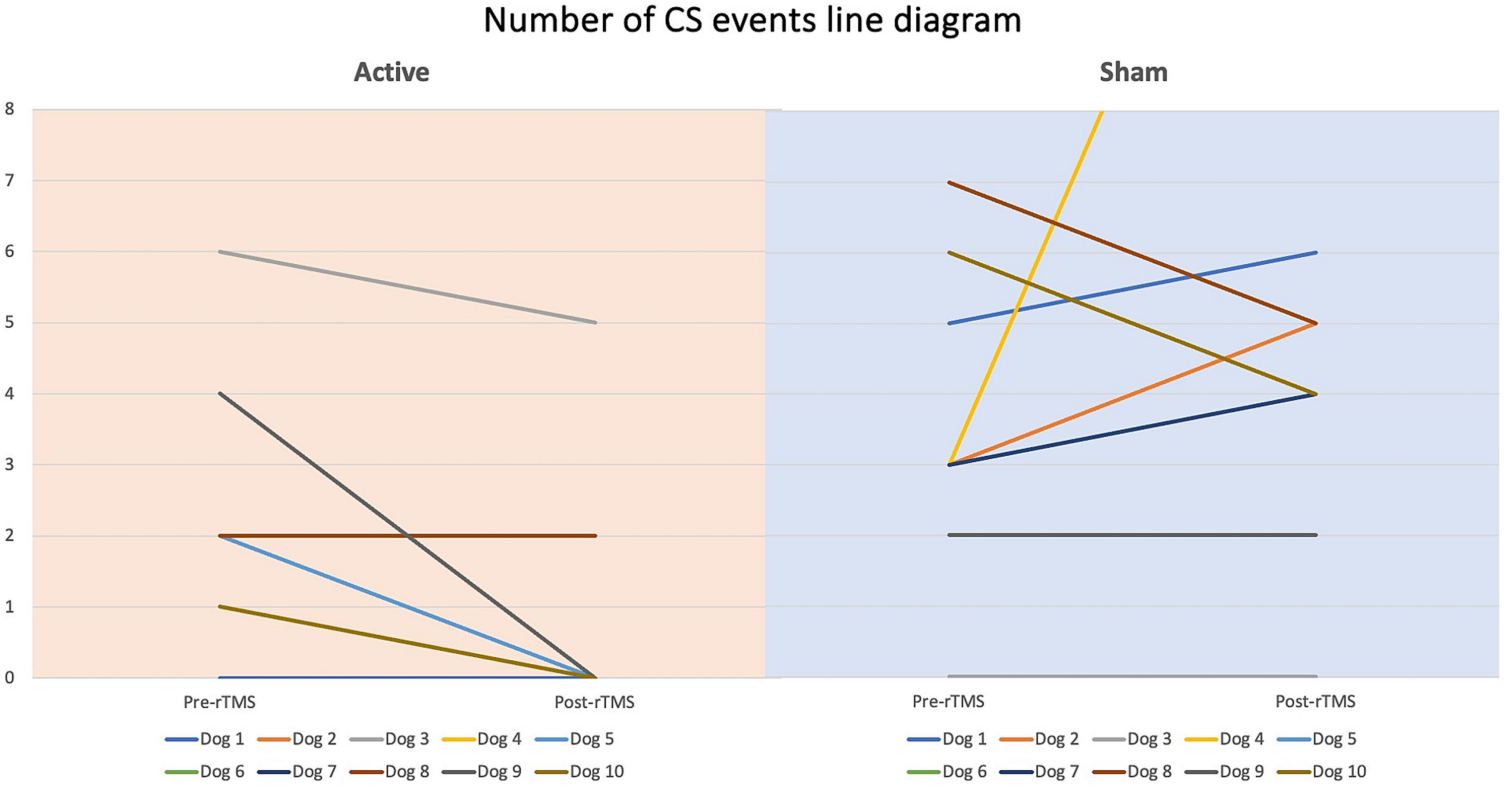
Line diagram showing the changes in number of CS events pre- vs. post-rTMS in both groups for each dog.

Dogs’ characteristics including breed, current age and age at seizure onset, sex as well as Tier diagnostic confidence level, seizure type, ASMs numbers and serum levels were included in Supplementary Table S1. IE and EUO was diagnosed in 90 and 10% of the dogs, respectively. Dogs were randomized to receive either active (*n* = 10) or sham rTMS (*n* = 10). One dog in the active and one in the sham group were euthanised at 2 and 3 weeks post-rTMS, respectively. At baseline, no significant differences were observed between the groups regarding disease characteristics and seizure frequency. Statistically significant differences were identified between the groups in median MSF (active, 8 [0–24]; sham, 17 [7–46]; *p* = 0.04), MSDF (active, 8 [0–24]; sham, 11 [6–23]; *p* = 0.04), number of CS (active, 10 [5–23]; sham, 16 [10–25]; *p* = 0.005). Results were also illustrated using box-plots (Figures 3–5) and line diagrams (Figures 6–8).

In the active group, the duration of the rTMS effect was assessed through extended follow-up data, which could be collected from five dogs beyond the initial three-month evaluation period. The median additional follow-up duration (excluding the three-month evaluation period) was 6 months (range, 4–10 months). Two dogs achieved seizure freedom during the evaluation period and were subsequently monitored for additional 10 and 5 months, respectively. In the former dog, seizure freedom persisted for an additional six-month period (9 months in total) before relapse occurred; however, the MSF remained below the pre-rTMS baseline for a further four-month period (13 months in total). In the latter dog, seizures recurred after the three-month evaluation period but remained at a frequency lower than the pre-rTMS MSF for an additional 5 months (8 months in total). In two other dogs, the extended follow-up periods were 6 and 8 months, respectively. During these intervals, not only did the MSF remain lower than the pre-rTMS baseline, but it continued to decrease further (nine and 11 months in total, respectively). In the final dog, the MSF remained below the pre-rTMS level for an additional 4 months (7 months in total). Overall, the median duration of the rTMS effect was 8 months (range, 3–13 months).

The median TMS coil output for the active group was 83% (mean, 85%; range, 70–100%). No remarkable alterations in the motor cortex threshold were noticed over the stimulation period.

## 4 Discussion

Our study investigated a three-day non-invasive neurostimulation protocol of rTMS as an adjunctive treatment for dogs with drug-resistant IE. Our findings indicate that active rTMS is safe and can reduce seizure frequency as well as prevent CS compared to sham. The duration of the rTMS effect ranged from 3–13 months; while a longer-lasting effect may be possible, this could not be determined due to the absence of follow-up data beyond these periods. Further exploration of rTMS protocols is needed to establish a more informed approach for the management of these challenging drug-resistant epilepsy cases.

The neurobiological effects of rTMS vary considerably among individuals (16). These effects are influenced by factors such as stimulation frequency, the number of stimuli per train, stimulation intensity, coil type, coil positioning, and duration of the stimulation. These parameters can be adjusted to target specific neuronal populations, enabling tailored cortical modulation for particular disorders (31). Despite the potential for such precision, a standardized set of optimal stimulation parameters for specific conditions, including epilepsy, has not yet determined (32).

Given that the pathophysiological hallmark of epilepsy is network disruption leading to cortical hyperexcitability, it is well-supported to pursue research on rTMS as a potential treatment alternative, particularly for drug-resistant epilepsy (17, 18). Moreover, rTMS is a non-invasive and straightforward technique to administer, further enhancing its appeal as a treatment option. Numerous human studies have investigated the effects of rTMS on epilepsy patients, reporting reductions in seizure frequency and/or epileptic discharges (20, 33–39). However, the efficacy of rTMS in decreasing seizures has not firmly established to date.

High-frequency rTMS (HF-rTMS; >5 Hz) is generally associated with facilitative effects on cortical excitability, whereas LF-rTMS (≤1 Hz) is known to decrease cortical excitability (40). There is a consensus that LF-rTMS reduces epileptic discharges and seizure frequency (10, 11, 13, 14, 20, 23, 32, 41–44). LF-rTMS applied for 15 to 30 min can effectively inhibit neural activity and reduce regional cortical excitability (45). This effect is believed to be mediated by the induction of neural plasticity mechanisms, such as long-term potentiation or depression, depending on the stimulation frequency (46). The impact of LF-rTMS can be robust and long-lasting (22, 45). While some reports indicate that LF-rTMS can significantly reduce seizure frequency and epileptic discharges, others have found no significant advantage over control treatments (33, 38, 47). These discrepancies can be attributed to the heterogeneous nature of the studies. Differences in the patient populations, including variations in disease etiology as well as inconsistencies in treatment parameters such as stimulation intensity, frequency, duration, and coil type, may explain the conflicting findings (41). Based on the concept that of LF-rTMS may exhibit inhibitory properties to the prosencephalon, a frequency of 1 Hz was utilized in our protocol with the aim to suppress the epileptogenic network. For the responders in our group, LF-rTMS effectively reduced seizure frequency. However, we did not compare it with HF-rTMS to assess potential differences. Additionally, other stimulation parameters likely influenced our response rates, and modifying these parameters might have led to different outcomes. Moreover, we did not employ advanced neuroimaging techniques such as electroencephalography-functional MRI, or spectroscopy to specifically evaluate cortical excitability in our clinical trial.

Repetitive TMS has been shown to induce structural changes in both the targeted brain regions and distant areas (48). While structural neuroplasticity is generally considered slower and less prevalent than functional plasticity (49), the exact timescale of these structural changes remains inadequately understood. Animal studies indicate that neurogenesis can occur within days, whereas more localized morphological alterations, such as the formation of new synapses and dendritic changes, may emerge over shorter timeframes (49–51). In our study, the variability in responses among individual animals could account for the observed differences in the onset of effects, with some subjects exhibiting immediate responses and others showing prolonged or delayed effects. Therefore, the therapeutic effects of rTMS may be long-lasting, but adequate time should be allowed post-treatment for neuroplastic changes to fully develop and optimize the clinical outcome. This consideration is essential before drawing definitive conclusions regarding the efficacy of the intervention in a particular case. Evaluating the impact solely over a short-term period may undervalue its effectiveness and could even result in false negative outcomes.

Apart from the rTMS frequency, other specific stimulation parameters, including the number of pulses and duration of treatment, show significant variability, especially across clinical trials involving human subjects (32). The persistence of rTMS effects beyond the active treatment phase is attributed to cumulative sessions (34, 35, 52, 53). An increased number of rTMS sessions has been associated with improved therapeutic outcomes (53). Although an increase in the number of pulses per rTMS session may be associated with improved therapeutic outcomes (54), this relationship is not universally observed (53, 55). The effect of LF-rTMS on cortical excitability is also dependent on the intensity of the stimulation, with studies indicating that higher intensities, e.g., > 70%, are significantly more effective in reducing seizure frequency compared to lower intensities (20, 34, 56). Indeed, findings from both our previous (14) and current studies indicate that applying LF-rTMS with a high number of pulses and coil intensity exceeding 70% over consecutive days can be effective in dogs with epilepsy. It is plausible that protocols involving LF-rTMS with an even greater number of pulses and higher maximum coil intensities applied over multiple days, e.g., stimulation sessions extending over several days or multiple sessions over a day, could potentially yield stronger and/or more prolonged antiseizure effects. However, this hypothesis requires validation through well-designed clinical trials in canine populations.

Last but not least, the geometry of the stimulation coil also plays a critical role in rTMS efficacy, influencing the depth and focal precision of the induced cortical currents (57). The figure-8 coil offers high focal precision for targeting specific cortical regions, while the circular coil distributes currents more diffusely across the cortical surface (18). In our research, given the generalized nature of IE in the subjects and the absence of a clearly defined epileptogenic zone, a circular coil positioned at the vertex was employed to achieve broad cortical stimulation. This approach aims to modulate excitability across the entire epileptic network, which may benefit patients with generalized epilepsy (31, 46, 58).

Notably, some evidence indicates that multifocal epilepsy may exhibit greater resistance to LF-rTMS treatment (59, 60). Research in humans has shown that the therapeutic effects of rTMS are often more pronounced in cases of neocortical epilepsy than in mesial-temporal lobe epilepsy. This disparity may be attributed to the deeper location of mesial-temporal structures within the brain, which poses challenges for the magnetic field to penetrate effectively, despite rTMS’s potential to indirectly influence deeper brain regions (20, 47). In our study in dogs, we were unable to confirm the type and foci of epilepsy, raising the possibility that the dogs showing a less favourable response may have had multifocal epilepsy or epilepsy involving deeper forebrain structures.

Although LF-rTMS is generally considered a low-risk clinical intervention, adverse effects such as transient headache, pain at the stimulation site, seizures, muscle contraction, and temporary tinnitus have been observed in humans (61). Additionally, there have been reports of rTMS activating epileptic foci in patients with medically intractable complex partial seizures (focal impaired awareness seizures), as well as inducing seizures in both healthy individuals and epilepsy patients (61–64). In our study, no adverse effects were observed.

While our findings highlight the potential value of rTMS as an adjunctive therapy for drug-resistant IE in dogs, the moderate sample size and the relatively short-term post-rTMS follow-up period limit the strength of the conclusions that can be drawn. Despite these limitations, the study offers novel insights into the management of drug-resistant epilepsy in dogs using a non-invasive and easily applicable neurostimulation technique, providing clinicians with another option to treat drug resistant epilepsy.

In conclusion, our study provides evidence supporting the antiseizure effects of LF-rTMS in dogs with drug-resistant epilepsy, demonstrating a significant reduction in seizure frequency and the number of CS in the actively treated group compared to the placebo group. Active LF-rTMS was likely effective in preventing CS in our population. Furthermore, our findings support the safety of this non-invasive neurostimulation technique in canine subjects. Given that specific stimulation parameters play a key role in determining the magnitude and persistence of antiseizure effects, the “one-fits-all” approach does not seem realistic in the field of neurostimulation. Specific TMS stimulation parameters may need to be individually tailored based on clinical or neurophysiological state and the severity of disease targeted. Therefore, personalized adjustments and optimization of rTMS protocols may further enhance long-term therapeutic outcomes. Larger-scale studies are warranted to evaluate various stimulation protocols in dogs, particularly those employing a high number of pulses and maximum intensity over several consecutive days, to confidently establish rTMS as an effective non-invasive neurostimulation treatment for drug-resistant epilepsy in dogs.

## Data Availability

The raw data supporting the conclusions of this article will be made available by the authors, without undue reservation.
