# Supplementary material for: Application of a novel three-day repetitive transcranial magnetic stimulation protocol for the treatment of drug-resistant epilepsy in dogs: single-blinded randomised sham-controlled clinical trial
Source: Front Vet Sci. 2025 Aug 6;12:1598311. doi: 10.3389/fvets.2025.1598311 (PMC12366470; doi:10.3389/fvets.2025.1598311)
Supplement: Supplementary file 1 [file Table_1.docx]

|  | **Active** (n=10) | **Sham** (n=10) |
| --- | --- | --- |
| Breed | Mixbreed (n=6)  Australian Shepherd (n=1)  Longhaired Collie (n=1)  Giant Schnauzer (n=1)  Siberian Husky (n=1) | Mixbreed (n= 2)  Australian Shepherd (n=2)  Saint Bernard (n=1)  Giant Spitz (n=1)  Samoyed (n=1)  Beagle (n=1)  Great Swiss Mountain Dog (n=1)  Mini Australian Shepherd (n=1) |
| Age (years) at seizure onset | Mean, 3.2; range, 1.0-7.0 | Mean, 2.6; range, 0.75-5.0 |
| Age (years) at inclusion | Mean, 4.5; range, 1.5-8.5 | Mean, 5.3; range, 2.0-6.5 |
| Sex and neuter status | Male intact (n=5)  Male neutered (n=4)  Female neutered (n=1) | Male intact (n=4)  Male neutered (n=3)  Female neutered (n=3) |
| Type of epileptic seizure | GTCS (n=8)  GTCS and FS (n=2) | GTCS (n=10) |
| Tier classification | TIER I (n=1)  TIER II (n=9) | TIER I (n=4)  TIER II (n=6) |
| Disease classification | IE (n=8)  EUO (n=1) | IE (n=10) |
| ASMs | PB/KBr/Imp (n=2)  PB/KBr/Leve (n=2)  PB/KBr (n=1)  PB/KBr/Imp/Leve (n=1)  PB/Imp (n=1)  PB/KBr/Imp/Zonisamide (n=1)  PB/KBr/Topiramate (n=1)  PB/Imp/Gabapentine (n=1) | PB/KBr (n=6)  PB/Imp (n=1)  PB/KBr/Imp (n=1)  PB/KBr/Imp/Leve (n=1)  PB/KBr/Leve/Felbamate (n=1) |
| ASMs serum levels | PB (mg/l): Mean, 30.1; range, 25.3-33.9  KBr (mg/ml): Mean, 1523; range, 1017-1986 | PB (mg/l): Mean, 31.7; range, 20.7-37.8  KBr (mg/ml): Mean, 1566; range, 1094-2148 |
| Cluster seizure events | 9 dogs (90 %) | 9 dogs (90 %) |

Table 1: Characteristics of the included dogs and disease

*ASMs, antiseizure medications; EUO, epilepsy of unknown origin; GTCS, generalised tonic clonic seizures; FS, focal seizures; KBr, potassium bromide; PB, phenobarbital; Leve, levetiracetam; IE, idiopathic epilepsy; Imp, imepitoin.*
